# Supplementary material for: Complexome profiling of the Chlamydomonas psb28 mutant reveals TEF5 as an early PSII assembly factor
Source: Plant Cell. 2025 Mar 17;37(6):koaf055. doi: 10.1093/plcell/koaf055 (PMC12164590; doi:10.1093/plcell/koaf055)
Supplement: koaf055_Supplementary_Data [file koaf055_supplementary_data.zip › psb28_interactive.zip!/plots/intensity/qualityPlots/peptides/_Cre13.g562501.t1.1.html]

### Cre13.g562501.t1.1

Trivial name:   
SeqCoverage [median]: 15.10  
Mass: 44.76 kDa  
  
 

| Sequence | MissedCleavage | Mass [Da] | wtIntensity | muIntensity | Score |
| --- | --- | --- | --- | --- | --- |
| AAGFSQAAAVGR | 0 | 1104.57 | 0.00 | 702.79 | 94.31 |
| AGDVLLLTKPLGVGAMTTAIK | 1 | 2068.20 | 0.00 | 912.71 | 60.76 |
| LEAPGAAAAAGTVVLQP | 0 | 1534.84 | 0.00 | 397.14 | 131.82 |
| VAAGAVPVMAPALALAQR | 0 | 1704.97 | 1191.83 | 1673.72 | 98.58 |
|  |  |  | 1191.8 | 3686.4 |  |
